# Initial Experience to Follow Lung Fluid Levels during Hemodialysis: A Possibility of Remote Dielectric Sensing-Guided Hemodialysis

**DOI:** 10.3390/jcdd9020057

**Published:** 2022-02-14

**Authors:** Hayato Fujioka, Teruhiko Imamura, Tsutomu Koike, Koichiro Kinugawa

**Affiliations:** The Second Department of Internal Medicine, University of Toyama, 2630 Sugitani, Toyama 930-0194, Japan; hfujioka@med.u-toyama.ac.jp (H.F.); tkoike@med.u-toyama.ac.jp (T.K.); kinugawa@med.u-toyama.ac.jp (K.K.)

**Keywords:** hypotension, congestion, hemodynamics, lung fluid, hemodialysis

## Abstract

Remote dielectric sensing (ReDS^TM^) is a novel technology that noninvasively quantifies lung fluid levels. Trends in ReDS values following hemodialysis remain uninvestigated. In a 64-year-old man with clinically stable hemodynamics, 2.7 L of fluid was drained during hemodialysis whereas the ReDS value remained almost unchanged (from 32 to 30%). In a 60-year-old woman with unstable hemodynamics, only 1.8 L of fluid was drained during hemodialysis, whereas ReDS value decreased considerably from 37 to 27%. Given our initial experience measuring ReDS values during hemodialysis, the ratio of fluid removal by hemodialysis between systemic plasma and lung fluid might vary in each patient. ReDS value might be a promising marker to determine the degree of fluid removal in addition to the conventional multidisciplinary index, particularly for those with unstable hemodynamics. The implications of ReDS-guided hemodialysis remain a future concern.

## 1. Introduction

A dry weight for hemodialysis is determined by multidisciplinary modalities including congestive symptoms, physical examination, echocardiography, and chest X-ray [1]. Of note, pulmonary congestion is key to the decision making of optimal dry weight settings. However, there are no gold standards to accurately assess lung fluid levels. Trends in lung fluid levels during hemodialysis remain uncertain. Insufficient fluid removal triggers fatal heart failure, whereas too much fluid removal triggers fatal intradialytic hypotension [2,3,4].

**Figure 1:**
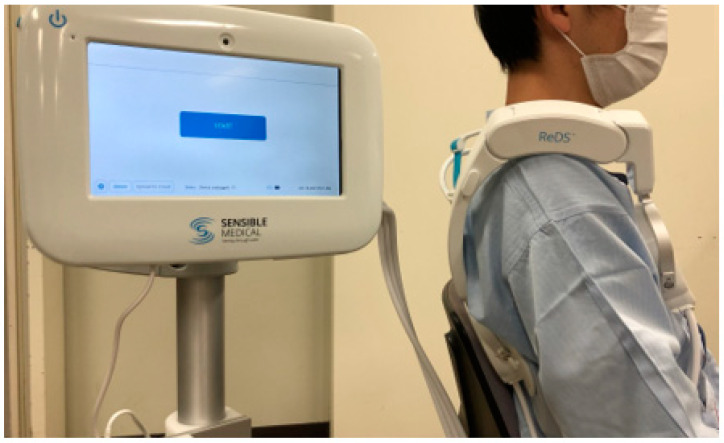
Remote dielectric sensing (ReDS^TM^) system with a controller and a sensor.

Recently, a remote dielectric sensing (ReDS^TM^) system, which is a non-invasive wearable device to quickly quantify the lung fluid level [5], has been introduced and become clinically available abroad (Figure 1) [6,7]. It emits low power electromagnetic signals across the thorax. The received signals, after passing through the tissue, indicate the lungs’ fluid content [5]. The ReDS value correlates with the degree of pulmonary congestion quantified by computed tomography in patients with and without heart failure, regardless of body size, although it is expected to be affected by electrodes, body habitus, fat content, and skin moisture [8,9]. The normal range of ReDS values suggested by the manufacturer is 20–35%. In previous clinical studies, aggressive treatment of hospitalized heart failure patients to keep ReDS below 35% after discharge reduced heart failure readmissions [6,7].

There are no reports on the use of ReDS in hemodialysis patients. Our institute initiated the use of this device for the first time in Japan before commercial use. ReDS values might give us valuable information regarding lung fluid levels and help us determine the degree of fluid removal. We will here present two patients in whom ReDS values were measured before and after hemodialysis.

## 2. Case Report

### 2.1. CASE 1 (ReDS Change from 32 to 30%)

Medical history: The first patient was a 64-year-old man with hereditary hemophilia type-A initiated hemodialysis seven years ago. His type-2 diabetes mellitus was well-controlled by oral medications. He had no cardiovascular diseases. His medications were as follows: amlodipine 5 mg/day, febuxostat 10 mg/day, evocalcet 2 mg/day, alfacalcidol 0.25 μg/day, precipitated calcium carbonate 3000 mg/day, lanthanum carbonate hydrate 2250 mg/day, and bixalomer 1500 mg/day.

**Figure 2:**
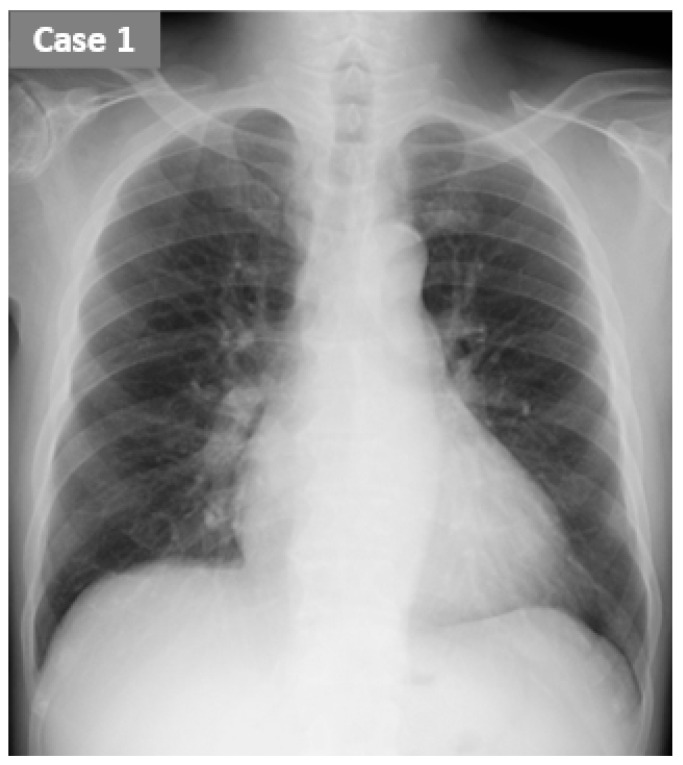
The chest X-ray just before hemodialysis in case 1.

Hemodialysis history: He had received 4 h hemodialysis using a 2.1 m^2^ polyether sulfone dialyzer without heparin support 3 times per week without any complications. His systolic blood pressure trended from 150 mmHg to 120 mmHg during hemodialysis. Laboratory data just before the hemodialysis are summarized in Table 1. Transthoracic echocardiography showed no remarkable findings, with a left ventricular ejection fraction of 76% without any obvious valve diseases. His dry weight was set at 50.0 kg. The mean increase in his body weight between hemodialysis was 2.1 kg. Just before hemodialysis, he had no signs of systemic congestion. A chest X-ray just before hemodialysis showed no pulmonary congestion (Figure 2). His dry weight was considered to be appropriate.

**Figure 3:**
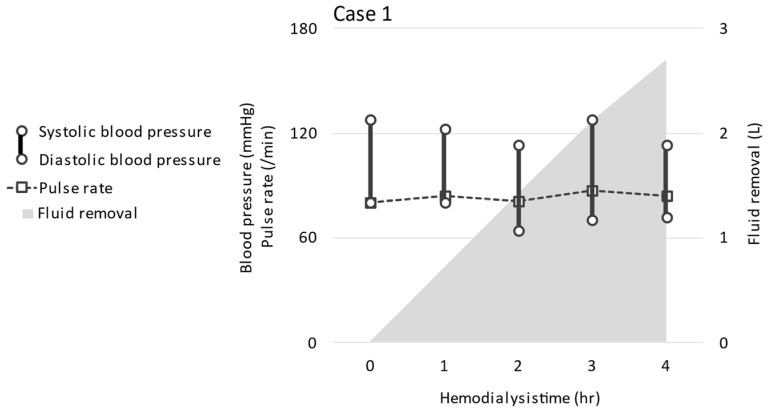
The trend of vital signs and fluid removal during hemodialysis in case 1.

Index hemodialysis and ReDS measurement: The patient’s body weight decreased from 52.5 kg to 49.8 kg by 2.7 L of fluid removal. ReDS value decreased slightly from 32% to 30% (normal range: between 20% and 35%). Blood pressure trended from 128/80 to 113/72 mmHg (Figure 3). Echocardiographic inferior vena cava diameter (inspiratory/expiratory) trended from 7/9 mm to 2/0 mm. Hematocrit increased from 34.5% to 40.9%. The intradialytic plasma volume decrease was calculated as 15.6% [10]. Trends in other parameters are summarized in Table 2.

### 2.2. CASE 2 (ReDS Change from 37 to 27%)

Medical history: The second patient was a 60-year-old woman with a history of type-2 diabetes mellitus, hypertension, and dyslipidemia. She received coronary artery bypass grafting five years ago. She initiated hemodialysis one year ago due to diabetic end-stage renal dysfunction. She was hospitalized due to worsening heart failure five months ago. She received a percutaneous coronary intervention due to unstable angina pectoris three months ago. Her medications were as follows: olmesartan 20 mg/day, aspirin 100 mg/day, clopidogrel 75 mg/day, atorvastatin 10 mg/day, and ezetimibe 10 mg/day, as well as bisoprolol transdermal patch 4 mg/day and insulin degludec 20 units per hemodialysis.

**Figure 4:**
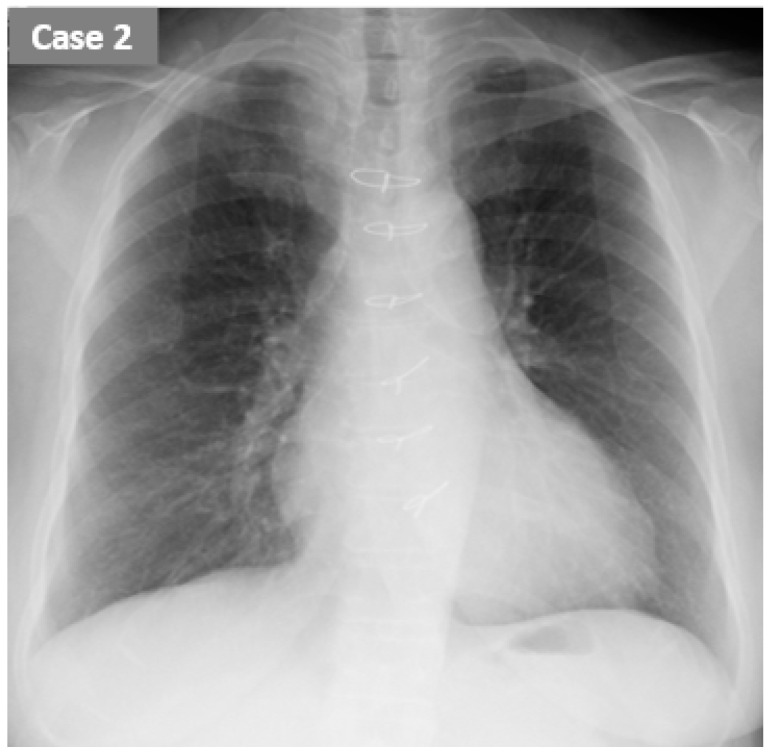
The chest X-ray just before hemodialysis in case 2.

Hemodialysis history: She received 4 h hemodialysis using 1.5 m^2^ polysulfone dialyzer with heparinization 3 times per week. Her systolic blood pressure decreased from 200 mmHg down to 100 mmHg during the hemodialysis despite etilefrine hydrochloride and amedinium methyl sulfate supports. Her laboratory data are summarized in Table 1. Her dry weight was set at 55.0 kg. Her mean increase in body weight between hemodialysis was as high as 3.2 kg. Transthoracic echocardiography showed a left ventricular ejection fraction of 60% without significant valve diseases. We assumed that the dry weight was appropriate given no peripheral edema and the chest X-ray cardiomegaly (Figure 4).

**Figure 5:**
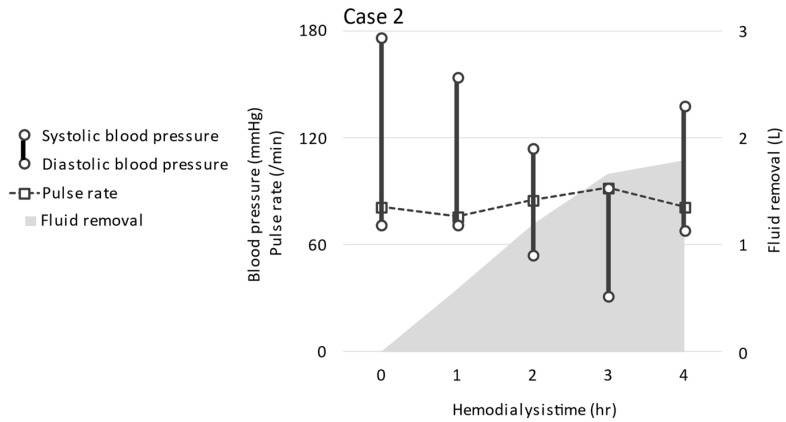
The trend of vital signs and fluid removal during hemodialysis in case 2.

Index hemodialysis and ReDS measurement: During the index hemodialysis, her body weight decreased from 57.3 kg to 55.8 kg by 1.8 kg of fluid removal. We could not achieve the targeted fluid removal given a decrease in systolic blood pressure down to 92 mmHg during the hemodialysis. ReDS value decreased from 37% to 27%. Blood pressure before and after the hemodialysis was 176/71 and 138/68 mmHg (Figure 5), respectively. Inferior vena cava diameter trended from 6/3 mm to 3/4 mm. Hematocrit trended from 32.4% to 32.7% (Table 2). The intradialytic plasma volume decrease was calculated as 0.9% [10].

## 3. Discussion

### 3.1. Lung Fluid Assessment

There have been no established methodologies to accurately assess the lung fluid levels, which should be one of the essential components for the fluid volume management in hemodialysis patients. Chest X-ray and computed tomography are conventional tools, but these require expert technique to assess. Human atrial natriuretic peptide is referenced to consider fluid valance during hemodialysis, but the optimal range remains uncertain [11]. Plasma B-type natriuretic peptide level can also be referenced in a general cohort, but this is inappropriately elevated in patients with end-stage renal disease [12].

ReDS is a novel device that can estimate lung fluid levels noninvasively, quickly, and easily [5]. ReDS value had a strong correlation with the degree of pulmonary congestion quantified by computed tomography using unique software [8,9]. We initiated the use of this device before Japanese commercial marketing, and this is an initial report in which ReDS values were measured during hemodialysis.

Both patients had relatively high ReDS values at baseline (32% and 37%) considering the manufacturer-suggested normal range (between 20% and 35%), although their chest X-ray seemed to have almost no pulmonary congestion. ReDS might be a useful tool to clarify the existence of sub-clinical pulmonary congestion that other modalities cannot find.

### 3.2. ReDS Measurement and Optimal Hemodialysis

In the case 1 patient, systemic plasma volume reduction was high, whereas ReDS reduction was low. On the contrary, in the case 2 patient, systemic plasma volume reduction was low, whereas ReDS reduction was considerable.

Given these findings, the ratio of fluid reduction during hemodialysis between systemic plasma and lung fluid might not necessarily be similar in all patients. Consistently, in case 2, despite the intradialytic plasma decrease being small, indicating an overhydrated status [13], the ReDS value decreased considerably. Such a classical index might not be appropriate to assess the fluid volume of every organ as with lungs. Detailed mechanism to explain such a difference requires further studies, but baseline fluid distribution might be key. The case 2 patient had heart failure with preserved ejection fraction and already had a great amount of lung fluid to be removed, despite a relatively hypovolemic status.

When the achievement of fluid removal to the level of dry weight is challenging due to unstable hemodynamics, as with the case 2 patient, ReDS measurement might become one of the practical indicators to set the degree of fluid removal. For example, in the case 2 patient, we attempted fluid removal aggressively to achieve the dry weight despite unstable hemodynamics. However, given the considerable reduction in lung fluid level, which was quantified by ReDS measurement, it might be permitted to weaken the degree of fluid removal for the prevention of worsening pulmonary congestion as well as intradialytic hypotension.

## 4. Conclusions

Although this is just a proof-of-concept, ReDS measurement might be a promising supportive tool to determine optimal dry weight. Optimal ReDS values that associate with favorable clinical outcomes remain the next concern. Clinical implications of ReDS-guided hemodialysis also remain a future concern.

## Figures and Tables

**Table 1:** Baseline characteristics.

		Case 1	Case 2
Total protein	(g/dL)	7.4	7.3
Albumin	(g/dL)	4.1	3.7
Urea nitrogen	(mg/dL)	76.4	74.7
Creatinine	(mg/dL)	11.53	8.35
Uric acid	(mg/dL)	4.3	7.5
Sodium	(mEq/L)	138	130
Potassium	(mEq/L)	5.2	6.1
Chloride	(mEq/L)	103	64
Calcium	(mg/dL)	8.8	8.1
Phosphorus	(mg/dL)	6.3	7.4
Glucose	(mg/dL)	99	185
C-reactive protein	(mg/dL)	0.31	0.67
White blood cells	(/μL)	6220	10,810
Red blood cells	(10^4^/μL)	386	343
Hemoglobin	(g/dL)	11.9	10
Hematocrit	(%)	35.6	30.6
Platelets	(10^4^/μL)	18.3	25.4
Glycoalbumin	(%)	15.7	26.6
Intact parathyroid hormone	(pg/mL)	125	233
β2-microglobulin	(μg/L)	40.9	
Kt/V for urea		1.51	1.45

Kt/V for urea was calculated by the Daugirdas method.

**Table 2:** Trends in clinical parameters during hemodialysis.

		Case 1		Case 2	
		Pre-HD	Post-HD	Pre-HD	Post-HD
Body weight	(kg)	52.5	49.8	57.3	55.8
ReDS value	(%)	32	30	37	27
Systolic blood pressure	(mmHg)	118	113	179	143
Diastolic blood pressure	(mmHg)	74	72	92	66
Pulse rate	(/min)	87	84	75	81
Inspiratory IVC diameter	(mm)	7	2	6	3
Expiratory IVC diameter	(mm)	9	0	7	4
Brain natriuretic peptide	(pg/mL)	47.3	-	189.7	-
Human atrial natriuretic peptide	(pg/mL)	-	19.4	-	51.9
Albumin	(g/dL)	3.9	4.7	3.9	4.2
Hematocrit	(%)	34.5	40.9	32.4	32.7
Intradialytic plasma volume decrease	(%)		15.6		0.9

Intradialytic plasma volume decrease = (Ht_post-HD_ − Ht_pre-HD_)/Ht_post-HD_ × 100. Ht, hematocrit; HD, hemodialysis; ReDS, remote dielectric sensing; IVC, inferior vena cava.

## Data Availability

No new data were created or analyzed in this study. Data sharing is not applicable to this article.

## References

[1] Flythe J.E., Chang T.I., Gallagher M.P., Lindley E., Madero M., Sarafidis P.A., Unruh M.L., Wang A.Y.-M., Weiner D.E., Cheung M. (2020). Blood pressure and volume management in dialysis: Conclusions from a Kidney Disease: Improving Global Outcomes (KDIGO) Controversies Conference. Kidney Int..

[2] Assimon M.M., Wenger J.B., Wang L., Flythe J.E. (2016). Ultrafiltration Rate and Mortality in Maintenance Hemodialysis Patients. Am. J. Kidney Dis..

[3] Burton J., Jefferies H.J., Selby N., McIntyre C.W. (2009). Hemodialysis-induced cardiac injury: Determinants and associated outcomes. Clin. J. Am. Soc. Nephrol..

[4] McIntyre C., Crowley L. (2016). Dying to Feel Better: The Central Role of Dialysis-Induced Tissue Hypoxia. Clin. J. Am. Soc. Nephrol..

[5] Amir O., Rappaport D., Zafrir B., Abraham W.T. (2013). A novel approach to monitoring pulmonary congestion in heart failure: Initial animal and clinical experiences using remote dielectric sensing technology. Congest. Heart Fail..

[6] Amir O., Ben-Gal T., Weinstein J.M., Schliamser J., Burkhoff D., Abbo A., Abraham W.T. (2017). Evaluation of remote dielectric sensing (ReDS) technology-guided therapy for decreasing heart failure re-hospitalizations. Int. J. Cardiol..

[7] Lala A., Barghash M.H., Giustino G., Alvarez-Garcia J., Konje S., Parikh A., Ullman J., Keith B., Donehey J., Mitter S.S. (2020). Early use of remote dielectric sensing after hospitalization to reduce heart failure readmissions. ESC Heart Fail..

[8] Amir O., Azzam Z.S., Gaspar T., Faranesh-Abboud S., Andria N., Burkhoff D., Abbo A., Abraham W.T. (2016). Validation of remote dielectric sensing (ReDS) technology for quantification of lung fluid status: Comparison to high resolution chest computed tomography in patients with and without acute heart failure. Int. J. Cardiol..

[9] Imamura T., Gonoi W., Hori M., Ueno Y., Narang N., Onoda H., Tanaka S., Nakamura M., Kataoka N., Ushijima R. (2021). Validation of Noninvasive Remote Dielectric Sensing System to Quantify Lung Fluid Levels. J. Clin. Med..

[10] Steuer R.R., Leypoldt J.K., Cheung A.K., Harris D.H., Conis J.M. (1994). Hematocrit as an indicator of blood volume and a predictor of intradialytic morbid events. ASAIO J..

[11] Hasegawa K., Matsushita Y., Inoue T., Morii H., Ishibashi M., Yamaji T. (1986). Plasma levels of atrial natriuretic peptide in patients with chronic renal failure. J. Clin. Endocrinol. Metab..

[12] Goei D., Schouten O., Boersma E., Welten G.M., Dunkelgrun M., Lindemans J., van Gestel Y.R., Hoeks S.E., Bax J.J., Poldermans D. (2008). Influence of renal function on the usefulness of N-terminal pro-B-type natriuretic peptide as a prognostic cardiac risk marker in patients undergoing noncardiac vascular surgery. Am. J. Cardiol..

[13] Rodriguez H.J., Domenici R., Diroll A., Goykhman I. (2005). Assessment of dry weight by monitoring changes in blood volume during hemodialysis using Crit-Line. Kidney Int..

